# The TPP Is Dead, Long Live the TPP? A Response to Recent Commentaries 

**DOI:** 10.15171/ijhpm.2017.12

**Published:** 2017-01-28

**Authors:** Ronald Labonté, Ashley Schram, Arne Ruckert

**Affiliations:** ^1^Canada Research Chair, Globalization and Health Equity, Faculty of Medicine, School of Epidemiology, Public Health and Preventive Medicine, University of Ottawa, Ottawa, ON, Canada.; ^2^School of Regulation and Global Governance, Australian National University, Canberra, Australia.; ^3^Faculty of Medicine, School of Epidemiology, Public Health and Preventive Medicine, University of Ottawa, Ottawa, ON, Canada.

One of President Trump’s first actions on assuming office was to formally withdraw the United States from the Trans-Pacific Partnership (TPP), ironically an agreement driven more by American business interests than by those of the other 11 signatory countries. But the issues raised in our health impact assessment (HIA) of the TPP,^1^ and the insightful commentaries it generated, have not died alongside the agreement. Regardless of the unpredictability of global politics during the Trump presidency, especially in terms of global trade relationships, the importance of ongoing analyses of the health impacts of trade has not abated. If anything, as several commentators note, it requires expansion, not diminishment.

## From Probable to Actual Outcomes

Blouin,^2^ while noting how trade negotiators still tend to ignore health cost externalities in crafting new agreements, makes the important point that what is needed now are *ex post* analyses of how the 400 trade deals worldwide have affected health, both directly, and via social determinants of health pathways. Although there have been analyses of the impacts of such agreements on the price of medicines, studies on broader health determinants (such as poverty or income distribution) have been lacking apart from generic measures of economic integration, of which trade treaties themselves are only one component. The same point was raised by Walls et al^3^ who, while welcoming our *ex ante* assessment, recognize the need for *ex post* studies of actual rather than only potential impacts. We agree; and our group at this moment is proposing just such a study, using a novel database that quantifies the depth of liberalization within trade agreements to examine how a number of key health outcomes and public policy variables are affected. The policy component is important for two reasons. First, as Walls et al comment, we need to understand better the policies that might mitigate trade’s health-harmful impacts. Second, as Blouin underscores, we need better evidence on the role of such agreements (notably investment protection provisions) in creating ‘regulatory chill’ – a topic we have begun to examine (with a paper presently under review) but which we agree is one requiring more empirical analysis. Have trade and investment agreements simply become another tool in the regulatory capture of the state by powerful transnationals?

## Investor State Dispute Settlement: Eliminate or Reform?

Lencucha’s^4^ commentary, focusing on ISDS (investor state dispute settlement) provisions within the TPP, would answer Blouin’s question with a powerful affirmative. The litany of what Lencucha calls the ‘nefarious use’ of ISDS continues with little abatement. The TPP, of course, is not the only agreement to have such provisions, controversies around which have stalled (and almost completely undone) the Comprehensive Economic and Trade Agreement (CETA) between Canada and the European Union. CETA has become the testing ground for ISDS reform, with the Trump election knocking the US/EU ‘Trans-Atlantic Trade and Investment Partnership’ (or TTIP) off the radar. In the name of ‘legal scrubbing,’ Canada and the EU actually renegotiated the text of its original ISDS provisions post-signing, creating something called an ‘Investment Court System’ (ICS).

As the commentary by McKee and Stuckler^5^ argue, the ICS improves on some of the procedural and interest-conflict provisions of ISDS, including greater transparency and a tightening of the bases upon which a dispute can be launched. That public health and broader civil society activism forced these ISDS reforms constitutes a small but important progressive step. The ICS’s ‘court system’ of ‘judges,’ however, has been rebuked by parts of the European legal community^6^ and, more fundamentally, these ISDS reforms still fail to address the substantive question: Why should foreign investors be given greater rights than domestic investors or citizens in challenging government regulations that they think compromises the value of their investment? We may be less sanguine than Lencucha in thinking that abandoning ISDS completely would incentivize the strengthening of domestic court systems to avoid a kleptocratic state seizure of foreign assets. But the history of the use of ISDS suggests that it has not improved foreign investment in developing countries with weak judiciaries (its putative *raison d’être*) and has become, instead, a game played between lawyers, hedge funds and transnational corporations based in high-income countries against governments regardless of the impartiality of their domestic court systems. The ICS may slow the game down somewhat, but will not prevent it entirely, with an early analysis of its provisions finding that they would not have been effective against a number of recent and highly controversial ISDS disputes.^7^

## Broadening the Scope of Health Impact Analyses

Focusing less on what we did report on, McNamara^8^ points out some of the limitations in our assessment, such as insufficient attention to how certain provisions might potentially work for health benefits, and lack of detailed analysis of the implications of the TPP on employment. We agree on these points, even collaborating with her on their elaboration.^9^ We also share her concern that a general shortcoming of HIAs of trade and investment treaties is their preoccupation with traditional (if nonetheless important) health topics, such as unhealthy commodities, and drug prices or health services, while ignoring arguably more important health determinants, such as employment and environment. In her call for more attention to these ‘broader determinants of health’ McNamara also queries the limitations of HIA as a tool for future analyses of the trade and health nexus. We are partly in agreement, as our own study adopted the broad structure of HIA but used multiple methods that, ultimately, created a synthetic analysis of the agreement’s probable health effects. Our study was less about being an HIA per se than using appropriate methods to address the questions we had posed, even if the methods and findings were packaged as an ‘impact assessment.’

In that sense, we are sympathetic to the critique made by Muntaner and Mahabir^10^ of our HIA’s ‘textualism’ and ‘pragmatic approach.’ Much of our related work to the HIA follows some of the methodological innovations that they suggest reside in a scientific realist approach, including theory testing and use of multiple methods to make the ‘black box’ of policy formulation transparent’ including the role of ‘ideology, resistance, and political power’ in policy outcomes. Perhaps more than these two commentators, however, we are also drawn to the usefulness of constructivist theory in understanding how these same three threads manifest as agreement or conflict in differing economic contexts, which forms the core of a related study on health and foreign policy in which we are actively engaged.^11^ But we share their same core argument that treaties such as the TPP embody in their design, text and probable impacts the interests of those economic elites who play a major role in their development, some evidence of which we offered elsewhere.^12-14^

## The Trans-Pacific Partnership: Semi-official Zombie Status

The points raised in these various commentaries, as well as our own analysis of the TPP, may seem moot given Trump’s summary execution of the agreement. But, along with the presumed demise of neoliberalism and the return of mercantilist protectionism that have been much heralded since the contrarian outcomes of the Brexit vote and the US presidential election, such conclusions may be premature. There are powerful capitalist interests in the United States that would be seriously harmed if that country’s borders did start to close to trade and the new Washington regime begin to shred the ‘new constitutionalism’ of its many global trade and investment treaties. Three-quarters of the Chinese products entering the United States, accounting for much of China’s touted trade-surplus and a key target in Trump’s populist cross-hairs,^15^ are actually American goods whose manufacturing had been outsourced to reduce labour and environmental protection costs.^16^ Many US industries similarly take advantage of immigrant labour from Mexico, and highly-subsidized American corn producers benefited from the North American Free Trade Agreement’s (NAFTA’s) evisceration of Mexico’s small-hold corn farming sector (and one of the motivators for mass migration northwards).^17^ The extent of global market integration achieved by neoliberalism’s forty year-run is different from that of the earlier era of economic globalization in the late 19th century to which our present time is frequently equated (including how its collapse ushered in two world wars). Today’s vertically integrated production chains and hypermobile capital will make it much harder to unravel or restrain economic integration.

This is not to say that a Trump administration would necessarily want to do so. Shortly after winning the election, Trump referred to a strategy of creating new bilateral trade deals to replace the ones he campaigned against. How different would these be from the now defunct TPP? Would they really exclude ISDS? Reduce the protectionism of expanded intellectual property rights? Weaken US oversight of other countries’ regulatory regimes? Have stronger labour and environmental protection measures? Improbable, to say the least, especially given that a recently published text-as-data analysis of the TPP found it to be overwhelmingly drawn from prior US trade agreements that reflected US economic and political interests.^18^ Just as several of the original TPP countries have carried on with their own ratification of the treaty, indicating support for its economic policies that could be resurrected in other agreements, the new US administration might simply export what it likes about the TPP into other trade negotiating venues while appearing to have scrapped the deal.

The TPP may yet prove to be a zombie, continually rising from the dead in only vaguely altered form. The real possibility of this renders our original analysis of the health impacts of the agreement, and the commentaries offered by colleagues in response, important points for ongoing public health lobbying efforts. As Thow and Gleeson^19^ emphasize, this is especially so since the new uncertainty of a (post?)-neoliberal era offers us an opportunity to argue more effectively for what a health-enhancing, socially just and ecologically-protective trading regime could (or should) look like. As they conclude, this will require greater trade policy and research capacity within public health and, more importantly and echoing a point consistent across all of the commentaries, a greater understanding of the political and economic power relations that set such trade and investment agendas in the first place.

## Ethical issues

Not applicable.

## Competing interests

Authors declare that they have no competing interests.

## Authors’ contributions

RL wrote the first draft; AS and AR contributed with revisions. All authors approved the final draft.

## Authors’ affiliations

^1^Canada Research Chair, Globalization and Health Equity, Faculty of Medicine, School of Epidemiology, Public Health and Preventive Medicine, University of Ottawa, Ottawa, ON, Canada. ^2^School of Regulation and Global Governance, Australian National University, Canberra, Australia. ^3^Faculty of Medicine, School of Epidemiology, Public Health and Preventive Medicine, University of Ottawa, Ottawa, ON, Canada.
